# Single-cell RNA sequencing reveals key molecular drivers and immune landscape in uveal melanoma: implications for targeted therapy and prognostic modeling

**DOI:** 10.3389/fimmu.2024.1493752

**Published:** 2024-11-20

**Authors:** Zeyu Song, Wenwen Shao, Zhikai Xiahou, Yue Xu, Xiaofeng Zhang

**Affiliations:** ^1^ Department of Ophthalmology, The Fourth Affiliated Hospital of Soochow University, Suzhou, China; ^2^ Shandong University of Traditional Chinese Medicine, Jinan, China; ^3^ China Institute of Sport and Health Science, Beijing Sport University, Beijing, China

**Keywords:** single-cell RNA sequencing, uveal melanoma, CITED1, immune cell infiltration, prognostic biomarkers

## Abstract

**Background:**

Uveal melanoma (UM), arising from melanocytes in the choroid, accounts for 3% to 5% of all melanocytic tumors and over 70% of intraocular malignancies. Despite effective local treatments, metastasis remains a significant challenge, with more than half of patients developing metastatic disease within ten years. Conventional therapies often yield poor outcomes, highlighting the urgent need for novel therapeutic strategies to enhance survival and prognosis for UM patients.

**Methods:**

We conducted a detailed analysis of the GSE139829 dataset, focusing on scRNA-seq data from eight primary UM patients and three with metastatic disease. Through clustering and marker gene expression analyses, we identified distinct subtypes of UM tumor cells and examined their transcriptional, metabolic, and intercellular communication profiles. We developed a novel prognostic model, PCOLCE TCs Risk Score (PTRS), centered on the C5 *PCOLC*E+ tumor cells, which was validated through *in vitro* functional assays. Additionally, we performed immune infiltration and metabolic pathway analyses to elucidate tumor-immune interactions and their clinical significance.

**Results:**

We identified eight distinct cell types in UM and classified tumor subpopulations into six subgroups. The C5 *PCOLCE*+ TCs subpopulation was highlighted as crucial in UM malignancy, demonstrating high differentiation potential and a significant role in tumor progression. CellChat analysis revealed substantial communication between C5 *PCOLCE*+ TCs and fibroblasts, suggesting their involvement in tumor growth and extracellular matrix remodeling. Metabolic pathway analysis indicated enhanced oxidative phosphorylation and glutathione metabolism in this subpopulation. Additionally, we developed a PTRS model based on C5 *PCOLCE*+ TCs, identifying *CITED1* as a high-risk gene that promotes UM cell proliferation, invasion, and migration *in vitro*.

**Conclusion:**

This study provides insights into UM metastasis via single-cell analysis, identifying C5 *PCOLCE*+ TCs as key malignancy drivers associated with oxidative phosphorylation and immune interactions. Our PTRS model highlights *CITED1* as a high-risk gene that promotes UM cell proliferation, paving the way for new prognostic models and therapeutic targets to enhance patient outcomes.

## Introduction

Uveal melanoma (UM) arises from melanocytes within the eye, predominantly in the choroid, with lesser involvement of the ciliary body and iris, representing 3% to 5% of all melanocytic tumors (1–3). While the incidence of cutaneous melanoma has escalated in recent years, the overall incidence of UM has remained relatively stable. Nevertheless, UM constitutes over 70% of intraocular malignant tumors, significantly jeopardizing patient health and survival (4–8). Current therapeutic approaches primarily encompass local interventions such as tumor resection, local radiotherapy, and ocular extraction, which effectively manage primary tumors (6, 9). Despite these measures, the risk of metastasis is alarmingly high, with more than half of patients experiencing metastatic spread within the first decade of diagnosis (10–12). Moreover, conventional treatments—including local embolization, radiofrequency ablation, surgical resection, and systemic chemotherapy—tend to yield poor outcomes after metastasis, with a median survival of approximately six months (13, 14). Consequently, identifying effective therapeutic strategies to inhibit UM metastasis is crucial for enhancing patient survival and prognosis.

Recent studies have increasingly focused on the immune microenvironment of UM. For instance, Qi Wan et al. examined the interplay between tumor stemness and the immune microenvironment, highlighting its implications for UM prognosis and the efficacy of immunotherapy (15). Notably, the interaction between the tumor immune microenvironment and tumor metabolism—particularly redox processes—plays a crucial role in regulating tumor growth, metastasis, and therapeutic responses. Maintaining redox homeostasis is essential for cellular physiological functions and survival, influencing key metabolic pathways such as oxidative phosphorylation and glycolysis (16). Compared to normal cells, UM tumor cells generally exhibit heightened levels of oxidative stress (17), while metastatic and circulating tumor cells demonstrate increased oxidative phosphorylation activity (18), suggesting a link between oxidative phosphorylation and tumor aggressiveness.

However, the role of oxidative stress in UM is complex and appears to be dualistic. Zhu et al. reported that oxidative stress can induce apoptosis in UM cells through the downregulation of SIRT1 (19), indicating that oxidative stress may exhibit antitumor effects in certain contexts. Conversely, Slater et al. identified elevated levels of the oxidative stress marker ATP5B as strongly correlated with poor prognosis and metastasis in UM (20), suggesting that oxidative stress might promote tumor progression under specific conditions. These contradictory findings underscore the need for further investigation into the distinct mechanisms of oxidative pathways in UM, as they may function differently across various settings.

Single-cell sequencing analysis has emerged as a pivotal tool in modern biomedical research, enabling the identification of distinct gene expression patterns and variants within individual cells of a tissue, thereby illuminating intercellular heterogeneity. This technique has gained traction in various disease studies, particularly oncology, offering critical insights into the tumor microenvironment, tumor progression, and therapeutic responses. In this investigation, we conducted a comprehensive analysis of single-cell data from UM, focusing specifically on tumor cells. Leveraging single-cell sequencing data, we examined the metabolic profiles, transcription factor networks, and intercellular communication dynamics among different UM tumor cell subclusters. Through CytoTRACE and Slingshot analyses, we identified a key subpopulation of UM tumor cells, designated C5 *PCOLCE*+ TCs, and developed a prognostic model for UM, referred to as PTRS. To validate the clinical applicability of this model, we selected *CITED1*, a prognostic gene within the PTRS framework, for *in vitro* experimentation. Our findings demonstrated that *CITED1* significantly influences the proliferation, migration, and invasion capabilities of UM cells, reinforcing its potential as a therapeutic target and underscoring the clinical predictive value of the PTRS model. Moving forward, we aim to further refine and validate this model to explore personalized therapeutic strategies targeting *CITED1*, thereby providing new avenues for treatment in UM patients.

## Methods

### Acquisition of UM single-cell data

The single-cell data for UM utilized in this study were sourced from the NCBI Gene Expression Omnibus (GEO) database (https://www.ncbi.nlm.nih.gov/geo/), specifically under the registration number GSE139829 (21), which comprises 11 UM samples (GSM4147091-GSM4147101). Additionally, relevant clinical data for UM were obtained from The Cancer Genome Atlas (TCGA) (https://portal.gdc.cancer.gov/) for further analysis.

### Processing and dimensionality reduction of UM scRNA-seq data

UM scRNA-seq data were processed and downscaled for clustering using the “Seurat” package (version 4.3.0) (22). To eliminate potential doublet cells, we employed the “DoubletFinder” R package (version 2.0.3) (23–26). Cells were filtered according to specific criteria to exclude low-quality data: (1) total gene transcript count (nCount) per cell ranging from 500 to 100,000; (2) number of transcribed genes per cell (nFeature) between 300 and 6,000; (3) mitochondrial gene ratio less than 25%; and (4) erythrocyte gene count ratio under 5%.

Using the “NormalizeData” function, we normalized the high-quality UM single-cell data to identify the top 2,000 highly variable genes, subsequently applying the “FindVariableFeatures” function (27–29). We then employed the “ScaleData” function (30, 31). Additionally, the “CellCycleScoring” function was utilized to evaluate cell cycle effects. Dimensionality reduction was conducted using the “RunPCA” function, with the first 30 valid principal components selected (32, 33). The “Harmony” R package (version 0.1.0) was employed to mitigate batch effects, followed by clustering analysis using the “FindNeighbors” and “FindClusters” functions (34, 35). Clusters were labeled based on existing literature, and a Uniform Manifold Approximation and Projection (UMAP) plot was generated for visualization (36, 37). The Ro/e algorithm was also implemented to assess tissue preferences among different cell clusters.

### Enrichment analysis of UM tumor subclusters

To effectively characterize the differentially expressed genes (DEGs) within each UM tumor subcluster, we employed the “FindAllMarkers” function in Seurat, coupled with the ‘ClusterProfiler’ R package for Gene Ontology Biological Process exploration (38). Additionally, we conducted Gene Set Enrichment Analysis (GSEA) for each UM tumor subcluster, focusing on Gene Ontology Biological Process (GOBP) enrichment entries and highlighting the pathways with the highest Normalized Enrichment Scores (NES).

### Trajectory analysis of UM tumor subclusters

We utilized CytoTRACE analysis (39) to evaluate and predict the relative differentiation status of UM tumor subclusters, with scores ranging from 0 to 1, reflecting a positive correlation with stem cell properties. Furthermore, we applied the “Slingshot” R package (version 2.6.0) (40) to infer the cellular lineage of the UM tumor subclusters. By employing the “getLineage” and “getCurves” functions, we mapped the differentiation trajectories of each subcluster, assessing the dynamic changes in gene expression over time. Based on the results of the pseudotemporal analysis, alongside previous findings, we identified key UM subclusters integral to tumor development.

### Metabolic analysis of UM tumor subclusters

We conducted a comprehensive analysis of the cellular metabolic pathway profiles of UM tumor subclusters using the R package scMetabolism. The top five metabolism-related pathways were visualized, allowing us to examine their distribution on the UMAP map as well as their expression across various tissue types.

### Identification of transcription factors and cellular communication analysis

The pySCENIC package (version 0.10.0) in Python (version 3.7) was employed to investigate the enrichment of transcription factors (TFs) and their regulatory activity within each UM subcluster. Initially, we applied the GRNBoost method to compute linkage weights between each TF and its potential target genes. This was followed by DNA sequencing analysis to identify possible direct binding targets. Subsequently, the regulatory activity in each cell was assessed using AUcell, allowing us to screen for the top five transcription factors with the highest activity, for which specificity scores were calculated and visualized.

To evaluate cellular communication among different UM subclusters, we utilized the “CellChat” R package (version 1.6.1) (41). This analysis inferred cellular interactions at the level of signaling pathways and receptor-ligand dynamics, with outgoing and incoming signaling patterns visualized to depict varying signal intensities across cell types.

### Construction of UM prognostic model and nomogram

To enhance clinical relevance, we constructed a novel prognostic model for UM using bulk RNA-seq data from UM patients. We conducted univariate Cox analysis on the top 100 candidate genes from key subgroups to identify those significantly associated with UM prognosis (42–44). To mitigate the risk of overfitting, LASSO regression analysis (45–48) was employed to determine the optimal λ value. And multivariate Cox analysis was performed to determine the coefficients for each gene according to the calculated formula: RISK SCORE = $\sum_{\text{i}}^{\text{n}}\text{Xi}\times \text{Yi}$ (X: coefficient, Y: gene expression level). Based on the optimal cut-off value of the PCOLCE TCs Risk Score, the UM cohort was categorized into high and low scoring groups, and Kaplan-Meier survival curves were generated to predict outcomes (49–51). Furthermore, we incorporated clinical factors such as age, race, tumor stage, and survival time to construct a nomogram for predicting overall survival (OS) using the “rms” R package, which was validated through ROC curve (52–54) and decision curve analyses (DCA).

### Immune infiltration and immune function analysis

We assessed immune infiltration in tumor tissues utilizing various algorithms, including ESTIMATE, CIBERSORT, EPIC, and Xcell. To investigate the correlation between immune cell populations and prognosis-related genes, we employed the “corrplot” R package (version 0.92) for correlation analysis. Additionally, immune cell abundance was quantified using the “CIBERSORT” R package (version 0.1.0) (55). To further elucidate the mechanisms of immune escape, we calculated Tumor Immune Dysfunction and Exclusion (TIDE) values across different groups.

### Functional enrichment analysis and mutational landscape analysis

After identifying differentially expressed genes (DEGs) across scoring groups, we performed Gene Ontology (GO) enrichment analysis (56, 57), encompassing Gene Ontology Cellular Component, Biological Process, and Molecular Function, to uncover potential differences in biological functions among the groups. We also utilized the “GSVA” R package to evaluate the biological properties and molecular distinctions of the groups, providing insights into their alterations in cellular behavior and function. Furthermore, we employed the “maftools” R package (58) to analyze the somatic mutation profiles of UM patients from the TCGA database, assessing the correlation between risk scores and tumor mutation burden (TMB) using the Spearman correlation test.

### Drug sensitivity analysis

We obtained the relevant drug sensitivity dataset from The Genomics of Drug Sensitivity in Cancer (GDSC) (https://www.cancerrxgene.org/). Utilizing the “pRRophetic” package (version 0.5) (59, 60), we calculated IC50 values to evaluate the drug sensitivity across different groups.

### Cell culture

MP65 and 92-1 cell lines were procured from the American Type Culture Collection (ATCC). Cells were maintained in RPMI-1640 medium supplemented with 10% fetal bovine serum and 1% penicillin/streptomycin. Cultures were incubated under standard conditions of 37°C, 5% CO2, and 95% humidity.

### Cell transfection

CITED1 knockdown was achieved using small interfering RNA (siRNA) constructs sourced from GenePharma (Suzhou, China). Transfection was conducted following the manufacturer’s instructions for Lipofectamine 3000 RNAi Max (Invitrogen, USA). Cells were seeded in 6-well plates at 50% confluence and transfected with either a negative control (si-NC) or CITED1 knockdown constructs (Si-CITED1-1 and Si-CITED1-2), employing Lipofectamine 3000 RNAiMAX for each transfection.

### Cell viability detection

Cell viability of the transfected MP65 and 92-1 cells was assessed using the CCK-8 assay. Cells were inoculated at a density of 5 × 10³ per well in a 96-well plate. Following incubation, 10 μL of CCK-8 solution (A311-01, Vazyme) was added to each well, and the plate was incubated in the dark at 37°C for 2 hours. Absorbance was measured at 450 nm using an enzyme-labeled instrument (A33978, Thermo) to evaluate cell viability on days 1, 2, 3, and 4. The average optical density (OD) values were calculated and represented in a line graph.

### Colony formation experiment

Transfected cells were seeded at a density of 1 × 10³ per well in a 6-well plate. Following a wash with PBS, the cells were fixed with 4% paraformaldehyde and stained with 0.1% crystal violet (Solarbio, China). Colony formation was then quantitatively analyzed.

### Transwell detection

Cells were inoculated into a 24-well plate chamber (Corning, USA), with or without matrix gel (BD Biosciences, USA). The cell suspension was added to the upper chamber of a Costar Transwell plate, while medium supplemented with serum was added to the lower chamber. After a 48-hour incubation in a cell incubator, the cells were fixed with 4% paraformaldehyde and stained with crystal violet. Migration and invasion abilities were subsequently assessed under a microscope.

### Wound healing test

Transfected cells were cultured in 6-well plates until they reached 95% confluence. A sterile 200 μL pipette tip was used to create linear scratches in the cell monolayer, followed by gentle washing with PBS. The culture medium was replaced, and images of the scratches were captured at 0 and 48 hours to measure the wound width.

### EdU proliferation detection

Transfected MP65 and 92-1 cells were seeded at a density of 3 × 10³ per well in a 6-well plate and incubated overnight. A 2× EdU working solution was prepared by adding a 10 mM EdU solution in serum-free medium, followed by a 2-hour incubation at 37°C. Cells were fixed with 4% paraformaldehyde for 30 minutes and treated with glycine (2 mg/mL) and 0.5% Triton X-100 for 15 minutes. Following fixation, the cells were incubated with 1 mL of 1× Apollo and 1 mL of 1× Hoechst 33342 for 30 minutes at room temperature. Cell proliferation was quantified using fluorescence microscopy.

### Statistical analysis

All analyses were conducted using R software (version 4.3.0). The Wilcoxon test and Pearson’s correlation coefficient were employed to assess significance between groups (*P< 0.05, **P< 0.01, ***P< 0.001).

## Results

### General overview of UM data

We obtained single-cell sequencing data for UM from the GEO database (GSE139829), comprising a total of 11 samples (GSM4147091-GSM4147101). After implementing quality control measures, we identified 87,529 high-quality cells derived from two tissue types: eight primary UM patients (T) and three metastatic UM patients (M) (**Supplementary Figure 1A**). These high-quality cells were classified using the Seurat framework, resulting in the identification of 39 Seurat clusters (**Supplementary Figure 1B**). Based on marker gene expression, these clusters were annotated into eight cell types: T_NK, endothelial cells (ECs), tumor cells, fibroblasts, photoreceptor cells, retinal pigment epithelial (RPE) cells, plasma cells, and myeloid cells (**Supplementary Figure 1C**). Notably, we focused on the tumor cell cluster, which predominantly originated from primary UM patients, although a subset was also derived from metastatic UM patients (**Supplementary Figure 1D**). The distribution of cell types across the two tissue sources was illustrated in **Supplementary Figure 1E**, revealing that 83.2% of tumor cells came from tumor tissues, while 67.1% were from metastatic tissues. Additionally, T_NK cells comprised 9.5% from tumor tissue and 18.0% from metastatic tissue. The top five genes expressed in each cell type are displayed in **Supplementary Figure 1F**, with *CD74, C1QA, C1QC, C1QB*, and *HLA-DRA* identified as the most prominent genes in the tumor cell cluster.

### Analysis of UM tumor subclusters

**Figure 1:**
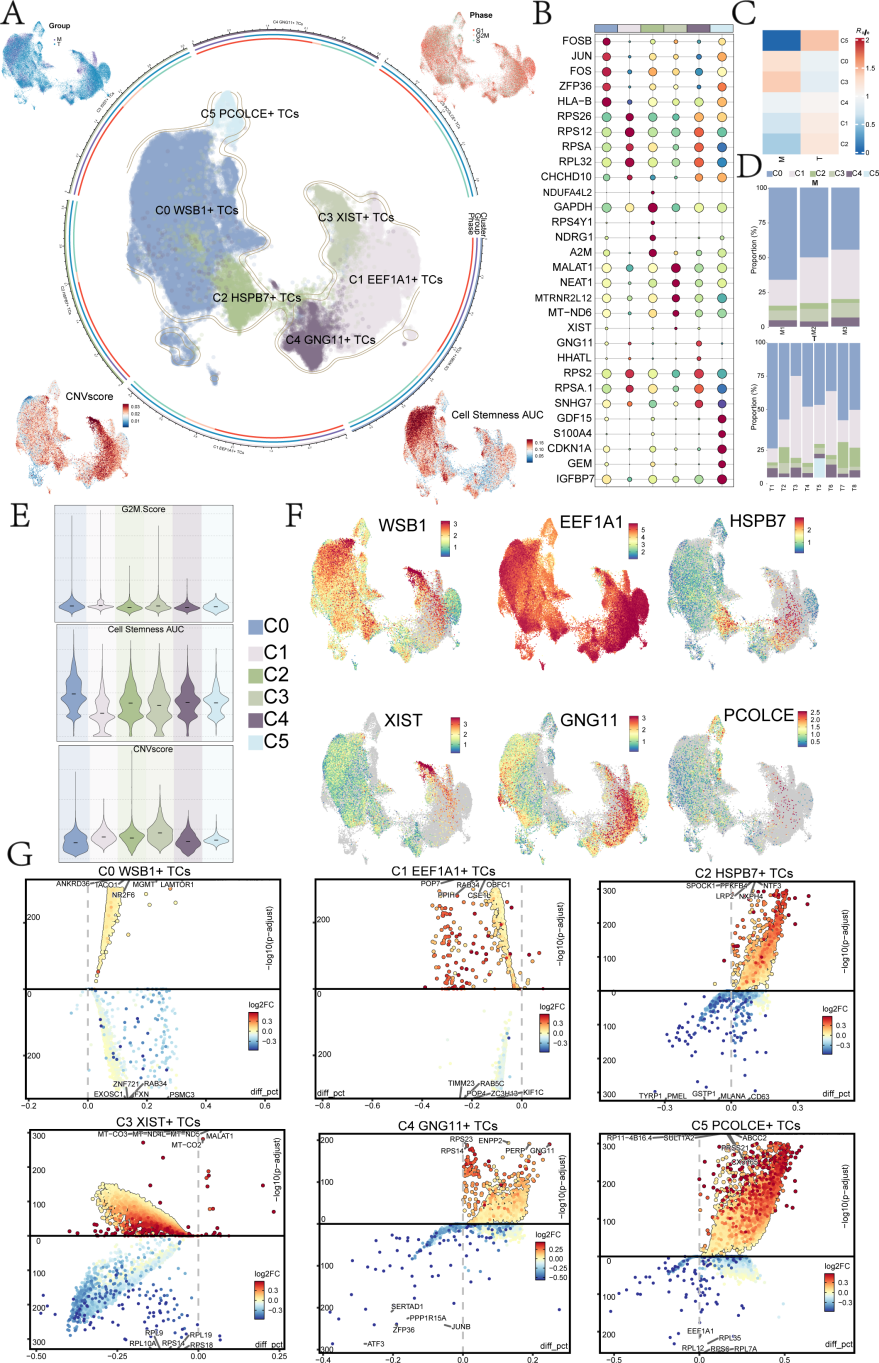
UM subpopulation analysis. **(A)** Tumor cells were reclassified into 6 UM cell subclusters based on marker genes, with UMAP plots in the middle illustrating their distribution. The peripheral UMAP plots displayed Group (M, T), Phase (G1, G2M, S), CNV Score, and Cell Stemness AUC. **(B)** A bubble diagram presented the top 5 marker genes of the 6 UM cell subclusters. **(C)** A heatmap illustrated the tissue preference Ro/e values for each UM cell subcluster. **(D)** The scale plots depicted the proportion of the 6 UM cell subclusters across different sample sources. **(E)** Violin plots demonstrated the specific expression levels of G2M Score, Cell Stemness AUC, and CNV Score for each UM cell subcluster. **(F)** UMAP plots displayed the distribution of named genes for each UM cell subcluster. **(G)** The volcano plots showed the top 5 DEGs for each UM cell subcluster.

To investigate the heterogeneity of tumor cells in UM, we conducted an in-depth analysis of the tumor cell clusters. We classified UM tumor cells into six distinct subclusters based on marker gene expression: C0 *WSB1*+ TCs, C1 *EEF1A1*+ TCs, C2 *HSPB7*+ TCs, C3 *XIST*+ TCs, C4 *GNG11*+ TCs, and C5 P*COLCE*+ TCs. The distribution of these subclusters was illustrated in the central UMAP plot of **Figure 1A**. Our analysis of the tumor cell origins (Groups M and T) and cell cycle phases (G1, G2M, and S) revealed that the C3 *XIST*+ TCs and C1 EEF1A1+ TCs subclusters were predominantly derived from Group T, with very few originating from Group M. Additionally, we assessed the copy number variation (CNV) core and Cell Stemness AUC for each UM subcluster (**Figures 1A, E**). The results indicated that C3 XIST+ TCs exhibited a higher CNV core, whereas C0 WSB1+ TCs displayed a greater Cell Stemness AUC. The InferCNV analysis results for the UM tumor cell subclusters were presented in **Supplementary Figure 2**.

The top five genes for each UM subcluster were identified and are shown in **Figure 1B**: C0 *WSB1*+ TCs (*FOSB, JUN, FOS, ZFP36, HLA-B*), C1 *EEF1A1*+ TCs (*RPS26, RPS12, RPSA, RPL32, CHCHD10*), C2 *HSPB7*+ TCs (*CHCHD10, NDUA4L2, GAPDH, RPS4Y1, NDRG1, A2M*), C3 *XIST*+ TCs (*MALAT1, NEAT1, MTRNR2L12, MT-ND6, XIST*), C4 *GNG11*+ TCs (*GNG11, HHATL, RPS2, RPSA.1, SNHG7*), and C5 *PCOLCE*+ TCs (*GDF15, S100A4, CDKN1A, GEM, IGFBP7*).

Analysis of tissue type preferences for each UM subcluster revealed that the C5 subcluster had the highest affinity for tumor tissue (**Figure 1C**). The specific proportions of sample sources for each subcluster were depicted in the scale diagram in **Figure 1D**, indicating that the C5 subcluster was primarily derived from T5, while the C0 subcluster originated from multiple patient samples. Furthermore, the analysis of the identified genes (*WSB1, EEF1A1, HSPB7, XIST, GNG11, PCOLCE*) highlighted that EEF1A1, associated with the C1 subcluster, was expressed across nearly all UM subclusters (**Figure 1F**).

### Functional enrichment analysis of UM subclusters

**Figure 2:**
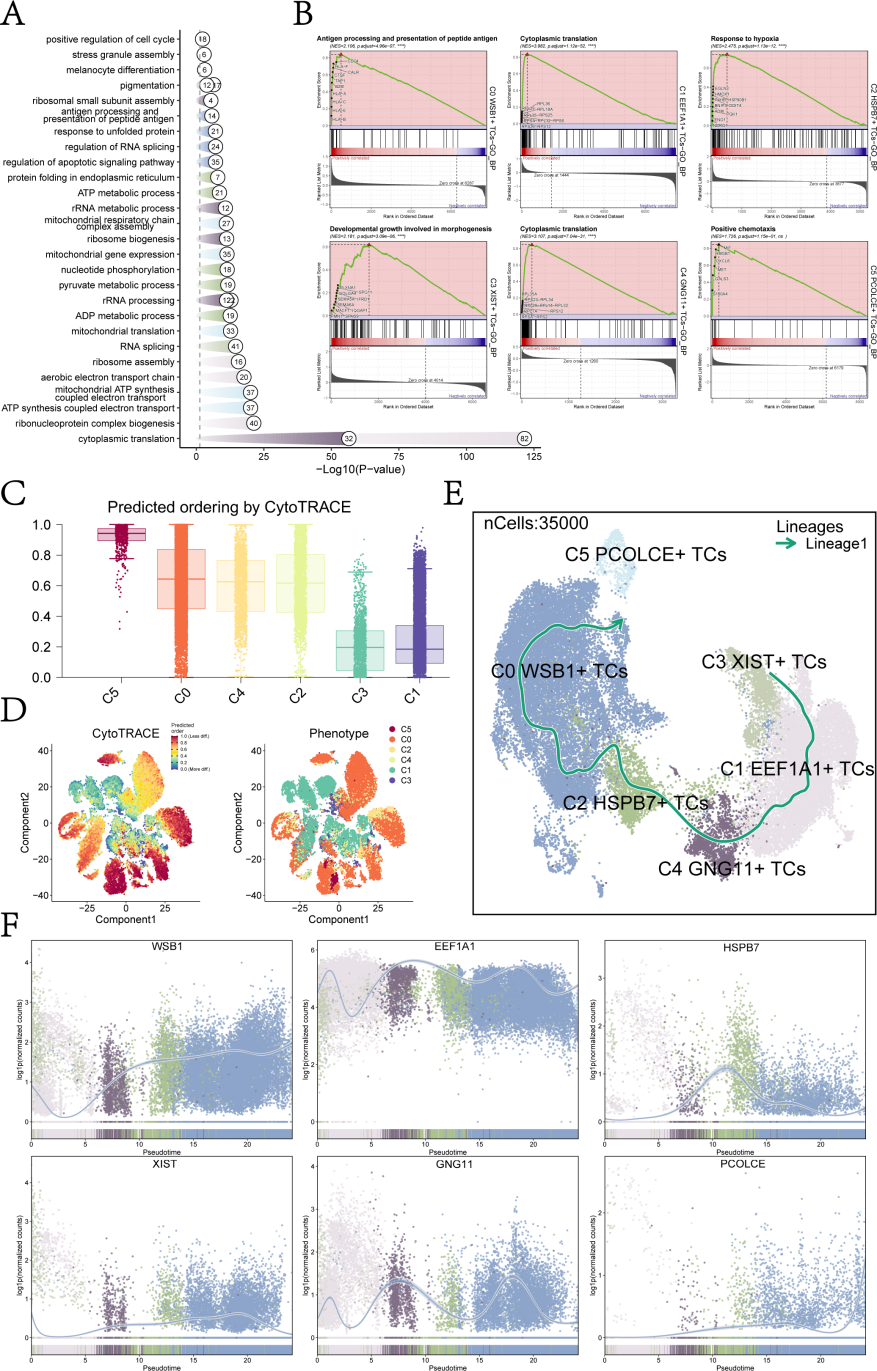
Subcluster enrichment analysis and pseudotime analysis. **(A)** Results of GOBP enrichment analysis were based on DEGs of each UM cell subcluster. **(B)** Results of GSEA enrichment analysis were presented for each UM cell subcluster, showing only the pathway with the highest NES value. **(C)** A bar graph depicted the CytoTRACE scores of each UM cell subcluster, with C5 exhibiting the highest score and C1 the lowest. **(D)** CytoTRACE results illustrated the degree of differentiation for each UM cell subcluster. In the left figure, dark green indicated greater differentiation (low stemness), while dark red indicated lesser differentiation (high stemness). The right figure represented different glioma subpopulations using various colors. **(E)** Developmental trajectories for each UM cell subcluster were determined using slingshot: C3 XIST+ TCs → C1 EEF1A1+ TCs → C4 GNG11+ TCs → C2 HSPB7+ TCs → C0 WSB1+ TCs → C5 PCOLCE+ TCs. **(F)** The scatter plots illustrated the trend of named genes for each UM subpopulation in relation to slingshot Lineage1 expression levels.

The top five DEGs for each UM subcluster were illustrated using volcano plots (**Figure 1G**), highlighting the most significantly up- and down-regulated genes in each subcluster. Subsequent analysis of these DEGs for GOBP enrichment aimed to identify biological processes that were overrepresented in each subcluster (**Figure 2A**). This analysis enhanced our understanding of the functional roles of DEGs within the various UM subclusters.

Building on the GOBP-enriched entries, we conducted GSEA for each UM subcluster to evaluate the significance of specific biological pathways and identify the entries with the highest NES in each subcluster (**Figure 2B**). The primary enriched biological processes were as follows: C0: antigen processing and presentation of peptide antigen; C1: cytoplasmic translation; C2: response to hypoxia; C3: developmental growth involved in morphogenesis; C4: cytoplasmic translation; and C5: positive chemotaxis. These findings indicate distinct functional specializations among the UM subclusters, reflecting their unique roles within the tumor microenvironment.

For instance, the enrichment observed in the C0 subcluster suggested a potential association with immune surveillance or immune evasion mechanisms, while the prominence of “cytoplasmic translation” in the C1 and C4 subclusters indicated heightened protein synthesis, likely linked to rapid cell proliferation or specific metabolic requirements. Overall, these results underscored the functional heterogeneity of UM subclusters, each possessing unique biological roles that might have influenced tumor behavior and therapeutic response.

### Analysis of proposed temporal trajectories of UM subclusters

To further investigate the heterogeneity within each tumor subcluster of UM, we employed various methods to analyze their developmental trajectories and differentiation potential. CytoTRACE analysis revealed that the C5 subcluster exhibited the highest CytoTRACE score, indicating greater developmental potential, while the C1 subcluster displayed the lowest score, reflecting a higher degree of differentiation and reduced developmental capacity (**Figure 2C, D**). These findings provided critical insights into the differentiation status and functional heterogeneity of UM cells.

We also conducted Slingshot analysis to elucidate the developmental trajectories of the UM subclusters. Lineage1 originated from C3 *XIST*+ TCs, progressing sequentially through C1 *EEF1A1*+ TCs, C4 *GNG11*+ TCs, C2 *HSPB7*+ TCs, and C0 *WSB1*+ TCs, ultimately culminating in C5 *PCOLCE*+ TCs (**Figure 2E**). Integrating these results with the CytoTRACE analysis, we identified the C5 *PCOLCE*+ TCs subcluster as a key player among UM malignant cells, positioned at the terminal stage of the developmental trajectory and possessing high differentiation potential. Therefore, we showed great research interest in the C5 *PCOLCE*+ TCs subcluster.

The naming genes for each UM subcluster within Lineage1 were displayed in **Figure 2F**. Notably, *EEF1A1*, the naming gene for the C1 subcluster, maintained high expression throughout the proposed temporal trajectory, whereas *PCOLCE*, associated with the C5 subcluster, consistently exhibited low expression levels, with a notable increase at the terminal stage. This suggested that the C5 *PCOLCE*+ TCs subcluster may play a critical role in the late stages of UM tumor progression, with changes in gene expression indicating its unique functions in tumor differentiation and potential metastasis. Given the upregulation of *PCOLCE* at the terminal stage, we hypothesized that it may contribute to regulating the tumor microenvironment, facilitating extracellular matrix remodeling, or interacting with other tumor-promoting factors to drive the progression of UM malignant cells.

### Metabolic analysis

**Figure 3:**
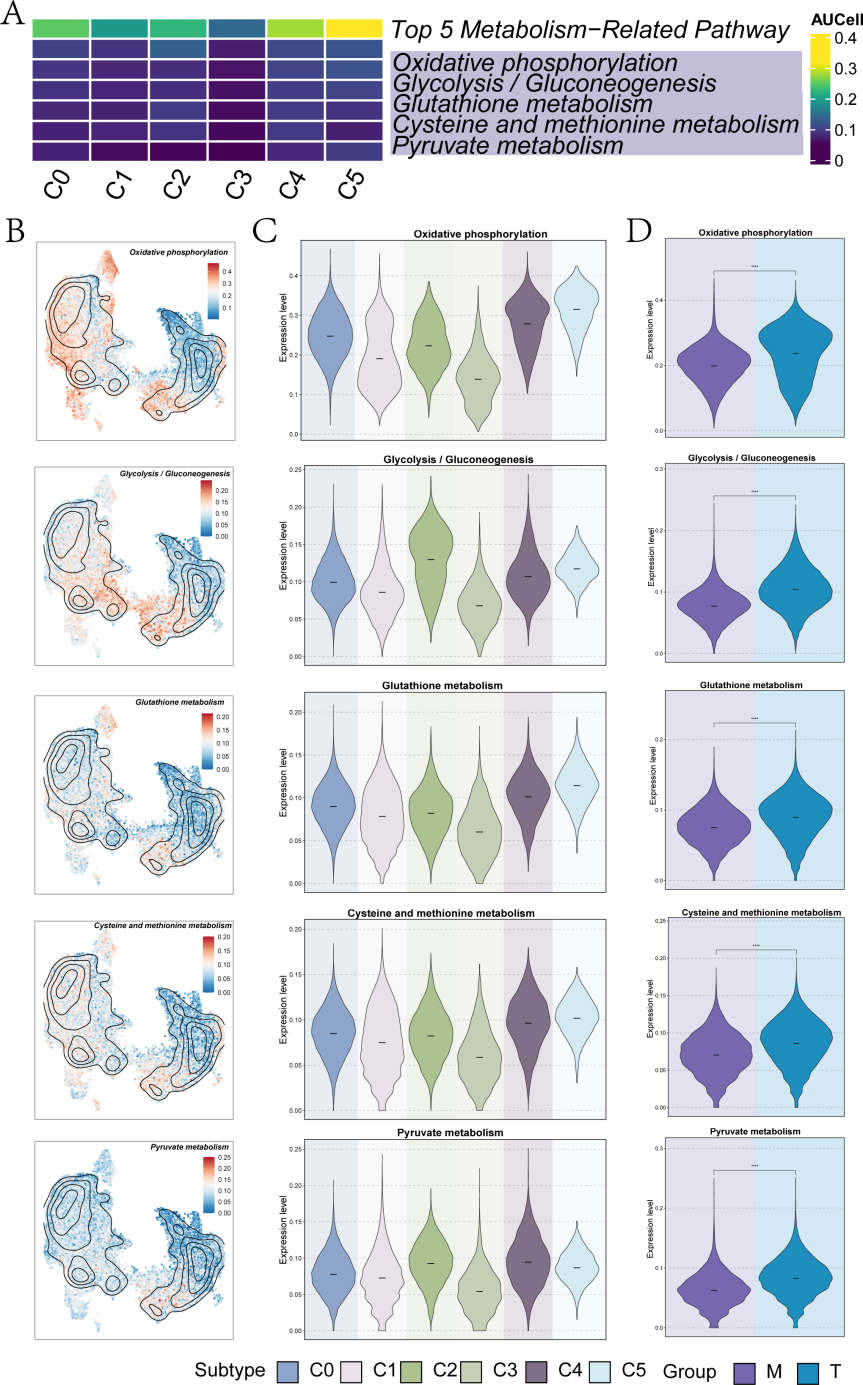
Metabolic analysis of UM subpopulations. **(A)** A heatmap illustrated the AUcells of each UM subpopulation in the top 5 metabolism-related pathways. **(B)** UMAP plots displayed the specific distribution of the top 5 metabolism-related pathways in each subpopulation. **(C)** The violin plots represented the specific expression levels of the top 5 metabolism-related pathways in each UM subpopulation. **(D)** The violin plots showed the specific expression levels of the top 5 metabolism-related pathways in each group (M and T). (****P< 0.0001).

The top five metabolism-related pathways of the UM subclusters were illustrated in **Figure 3A**, including oxidative phosphorylation, glycolysis/gluconeogenesis, and glutathione metabolism. The distribution and expression levels of these pathways across each UM subpopulation were presented in **Figures 3B** and **3C**. Notably, C4 *GNG11*+ TCs and C5 *PCOLCE*+ TCs exhibited elevated expression across all five metabolic pathways (**Figure 3B**). Among these, the C5 subcluster displayed particularly high expression levels in oxidative phosphorylation, glutathione metabolism, and cysteine and methionine metabolism when compared to other UM subclusters (**Figure 3C**). Additionally, the expression of these metabolic pathways across different tissue sources (T and M) was depicted in the violin plot of **Figure 3D**, revealing significantly higher expression levels in T tissues, with all results demonstrating statistical significance.

### Transcription factor analysis

**Figure 4:**
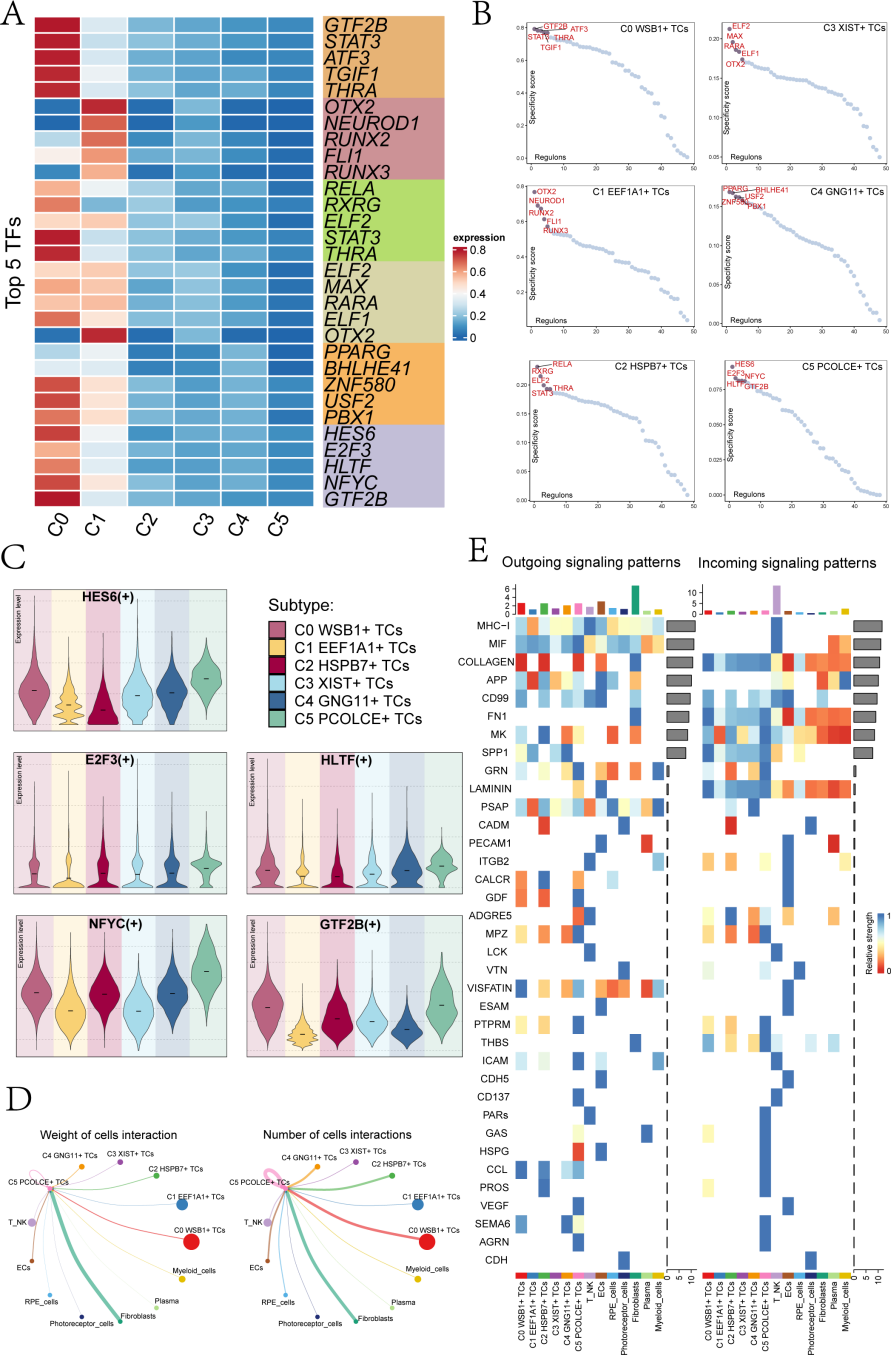
Transcription factor and cellular communication analysis of subgroups. **(A)** A heatmap displayed the top 5 TFs of the 6 UM cell subclusters. **(B)** UM subcluster transcription factors were ranked based on their Regulon specificity score (RSS). **(C)** Violin plots illustrated the top 5 TFs of the C5 PCOLCE+ TCs subcluster across each UM subcluster. **(D)** Circle plots represented the weight of cell interactions (left) and the number of cell interactions (right) between the C5 PCOLCE+ TCs subcluster and other subclusters. Thicker lines indicated a higher number of interactions and stronger interaction weight between the two cell types. **(E)** An overview was provided of outgoing and incoming signaling patterns.

To identify the core TFs within the UM subclusters, we conducted a SCENIC analysis. Utilizing pySCENIC, we inferred the gene regulatory networks associated with these subclusters. The top five TFs and their relative expression levels across each UM subcluster were presented in **Figure 4A**. The most active TFs identified were *GTF2B* in C0 *WSB1*+ TCs, *OTX2* in C1 *EEF1A*1+ TCs, *REL*A in C2 *HSPB7*+ TCs, *ELF2* in C3 *XIST*+ TCs, *PPARG* in C4 *GNG11*+ TCs, and *HES6* in C5 *PCOLCE*+ TCs. **Figure 4B** ranks these transcription factors based on their Regulon specificity scores (RSS). In the C5 *PCOLCE*+ TCs subcluster, the top five TFs identified were *HES6, E2F3, HLTF, NFYC*, and *GTF2B*, with their specific expression levels detailed in **Figure 4C**. Notably, *HES6* exhibited high expression in the C3 *XIST*+ TCs, C4 *GNG11*+ TCs, and C5 *PCOLCE*+ TCs subclusters.

### Cell communication analysis

Cell communication encompassed the capacity of cells to receive, process, and transmit signals, playing a crucial role in coordinating various biological activities such as development, differentiation, and inflammation. This interaction was primarily facilitated by ligand-receptor complexes, enhancing cell-to-cell communication. In our study, we employed the “CellChat” R package to quantitatively infer and analyze the communication networks among UM cells. The results revealed extensive communication links across different cell types, particularly focusing on the interactions between the C5 *PCOLCE*+ TCs subcluster and other clusters. Notably, the number and intensity of interactions between C5 *PCOLCE*+ TCs and fibroblasts were elevated, suggesting a significant signaling exchange between these cell types (**Figure 4D**). This robust interaction implies that C5 *PCOLCE*+ TCs may play a pivotal role in tumor progression and related biological processes, such as tissue remodeling and extracellular matrix regulation, through their communication with fibroblasts in the tumor microenvironment. **Figure 4E** illustrated the afferent and efferent signal strengths of all UM cell interactions, highlighting that nearly all UM subclusters are associated with CD99. Furthermore, within the incoming signaling patterns, the C5 *PCOLCE*+ TCs subgroup exhibited strong connections to CCL, SEMA6, LAMININ, and MK, underscoring the importance of these pathways in cell signal transduction.

### Construction and analysis of PTRS model

**Figure 5:**
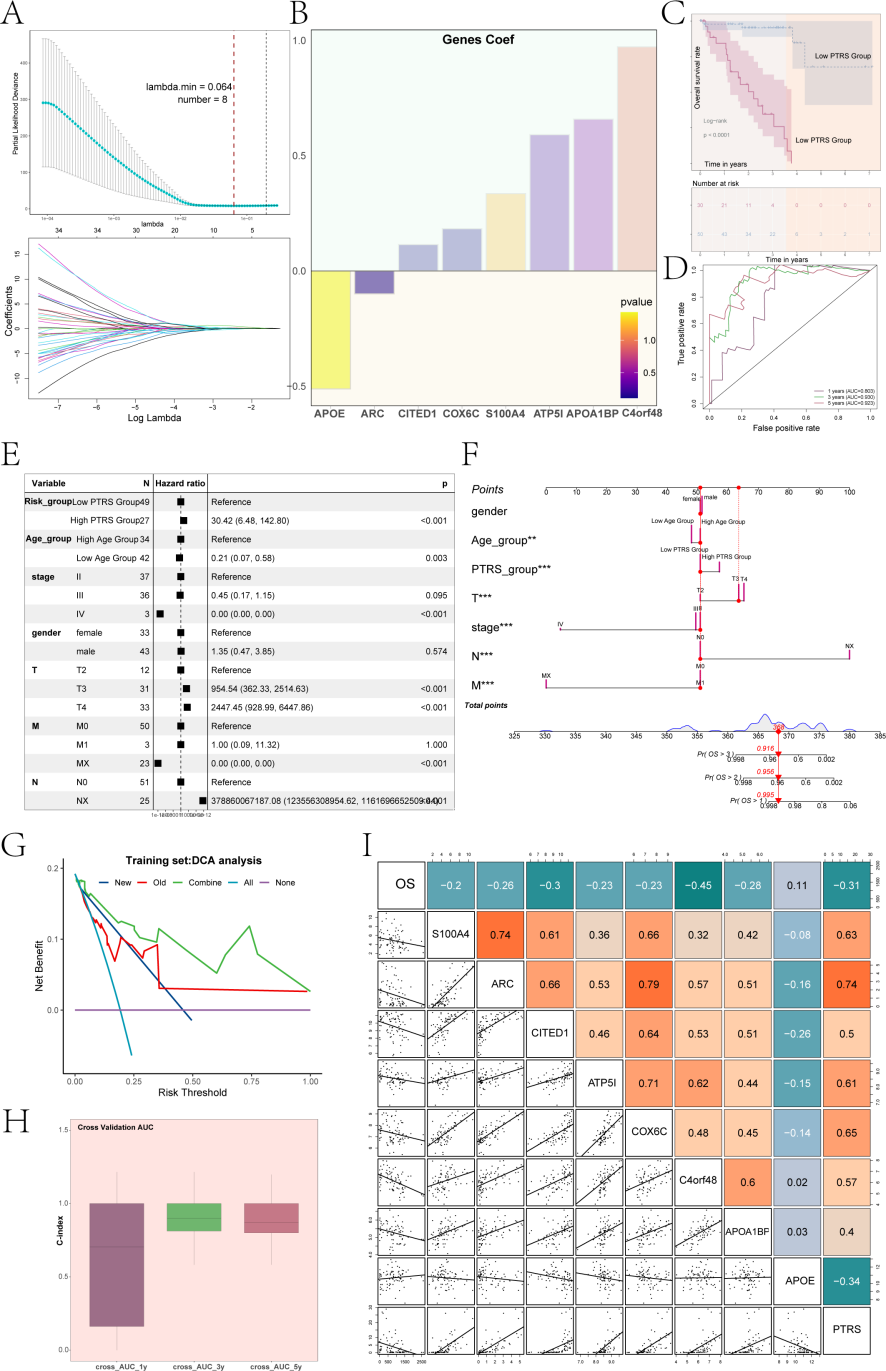
UM novel modeling and its correlation analysis. **(A)** Results of LASSO regression analysis indicated that, with lambda.min = 0.064, the constructed UM model was optimal, including a total of 8 genes. **(B)** A bar graph displayed the coefficients of UM prognosis-related genes along with their corresponding P-values. **(C)** Results of Kaplan-Meier survival analysis was shown for the low PTRS group and high PTRS group. **(D)** ROC curves of the PTRS model were presented, with AUC values of 0.803, 0.930, and 0.923 for 1 year, 3 years, and 5 years, respectively. **(E)** A forest plot illustrated the results of multivariate Cox regression analysis, indicating PTRS as an independent risk factor. **(F)** A nomogram was constructed by combining PTRS with clinical factors (race, age, staging). **(G)** DCA assessed the reliability of the new UM model. **(H)** The bar graph demonstrated the C-index of the predictive nomogram modeling. **(I)** A heatmap combined with a dot plot showed the correlation between OS, 8 prognostic genes of the PTRS model, and PTRS. **P< 0.01; ***P< 0.001.

To enhance clinical decision-making for UM and to gain deeper insights into patient prognosis, we developed a novel prognostic model centered on the key C5 *PCOLCE*+ TCs subgroup. We began by conducting univariate Cox analysis on the top 100 candidate genes from this subgroup to identify those with significant prognostic relevance (**Supplementary Figure 3**). Subsequently, we performed LASSO regression analysis on the selected prognostic genes, yielding a lambda.min of 0.064 and identifying eight crucial genes (**Figure 5A**): *APOE, ARC, CITED1, COX6C, S100A4, ATP5I*, *APOA1BP*, and *C4orf48*. Multivariate Cox regression analysis revealed that APOE acted as a protective gene, while the majority were associated with increased risk (**Figure 5B**).

Using the expression levels of these genes and their respective coefficients, we constructed a new UM prognostic model, termed the *PCOLCE* TCs Risk Score (PTRS). The calculation formula was defined as: *PCOLCE* TCs Risk Score (PTRS)= $\sum_{\text{i}}^{\text{n}}\text{Xi}\times \text{Yi}$ (X: coefficient, Y: gene expression level). Based on the optimal cutoff value of the PTRS, we categorized UM patients into Low PTRS and High PTRS groups to analyze survival outcomes. Consistent with our expectations, results indicated that the High PTRS group had a poorer prognosis (**Figure 5C**). Additionally, to evaluate the predictive capability of the PTRS model, we generated ROC curves, yielding AUC values of 0.803, 0.930, and 0.923 at 1, 3, and 5 years, respectively (**Figure 5D**), thereby demonstrating the model’s high sensitivity and specificity.

We conducted multivariate Cox regression analysis incorporating clinical factors alongside the PTRS to evaluate its potential as an independent risk factor. The analysis revealed a hazard ratio (HR) for PTRS of 30.42, with a 95% confidence interval (CI) of 6.48–142.80 and a P-value< 0.001 (**Figure 5E**). This finding underscored the significant association of PTRS with prognosis, suggesting its role as an independent prognostic indicator for UM patients and providing robust support for clinical decision-making.

To enhance prognostic accuracy, we developed a nomogram integrating clinical factors—such as gender, age, and tumor clinical stage (T, N, M)—alongside PTRS to predict overall survival (OS) for UM patients at 1, 3, and 5 years (**Figure 5F**). Both the PTRS subgroups and tumor clinical stage significantly influenced OS outcomes. Decision Curve Analysis (DCA) further evaluated the reliability of this UM prognostic model (**Figure 5G**), confirming its utility as a clinical decision support tool. The C-index for the nomogram model, displayed in **Figure 5H**, indicated predictive accuracy ranging from 0.5 to 1 for OS at 1, 3, and 5 years, demonstrating the model’s robust predictive ability across various time points. Notably, the C-index approached 1 over time, reinforcing its reliability for long-term prognostic assessments.

Additionally, **Figure 5I** illustrated the correlation between the eight prognostic genes within the PTRS model and both OS and PTRS, revealing that OS was negatively correlated with most of these genes. This comprehensive analysis highlighted the potential of the nomogram in providing valuable prognostic information for clinical practice.

### PTRS model correlation analysis

**Figure 6:**
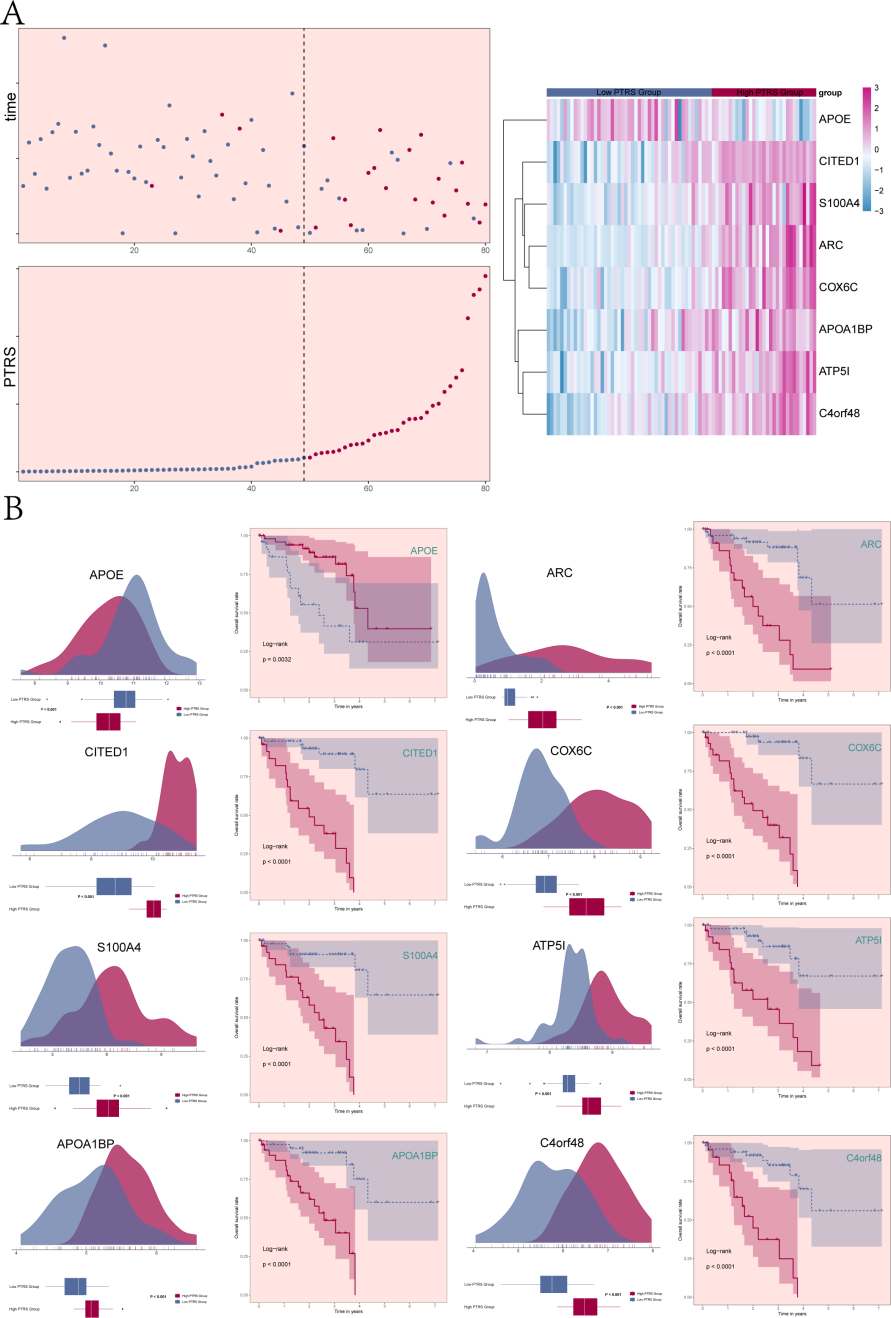
Correlation analysis of genes constituting PTRS. **(A)** Scatter plot and curve plot illustrated the survival/death status and PTRS levels across different PTRS groups over time (left). A heatmap displayed the distribution of 8 UM prognostic genes among the different PTRS groups (right). **(B)** The ridge plots combined with boxplots demonstrated the expression levels of the 8 genes constituting PTRS in different PTRS groups. Based on the optimal threshold of gene expression levels, samples were categorized into high and low expression groups. Kaplan-Meier survival curves revealed prognostic differences between the high and low expression groups of genes, with all results being statistically significant.

We investigated the survival and mortality status, as well as the levels of the PTRS, across the Low and High PTRS Groups over time, alongside the overall distribution of the eight prognostic genes within the PTRS model (**Figure 6A**). The specific distribution of these genes in different PTRS cohorts was illustrated using ridge plots combined with boxplots (**Figure 6B**). Notably, *APOE* exhibited higher expression in the Low PTRS Group, whereas the other seven genes (*ARC, CITED1, COX6C, S100A4, ATP5I, APOA1BP*, and *C4orf48*) were more highly expressed in the High PTRS Group, with all differences being statistically significant.

Based on the expression levels of the eight PTRS model genes, the UM patient cohort was stratified into high and low expression groups for each gene (**Figure 6B**). The analysis revealed that the High APOE Group experienced better survival outcomes compared to the Low APOE Group, with this difference also being statistically significant. In contrast, the remaining seven PTRS model prognostic genes were associated with poorer prognosis in the High expression group, further establishing APOE as a protective factor while indicating that the other genes serve as risk factors.

### Analysis of immune infiltration and immune function in different PTRS groups

**Figure 7:**
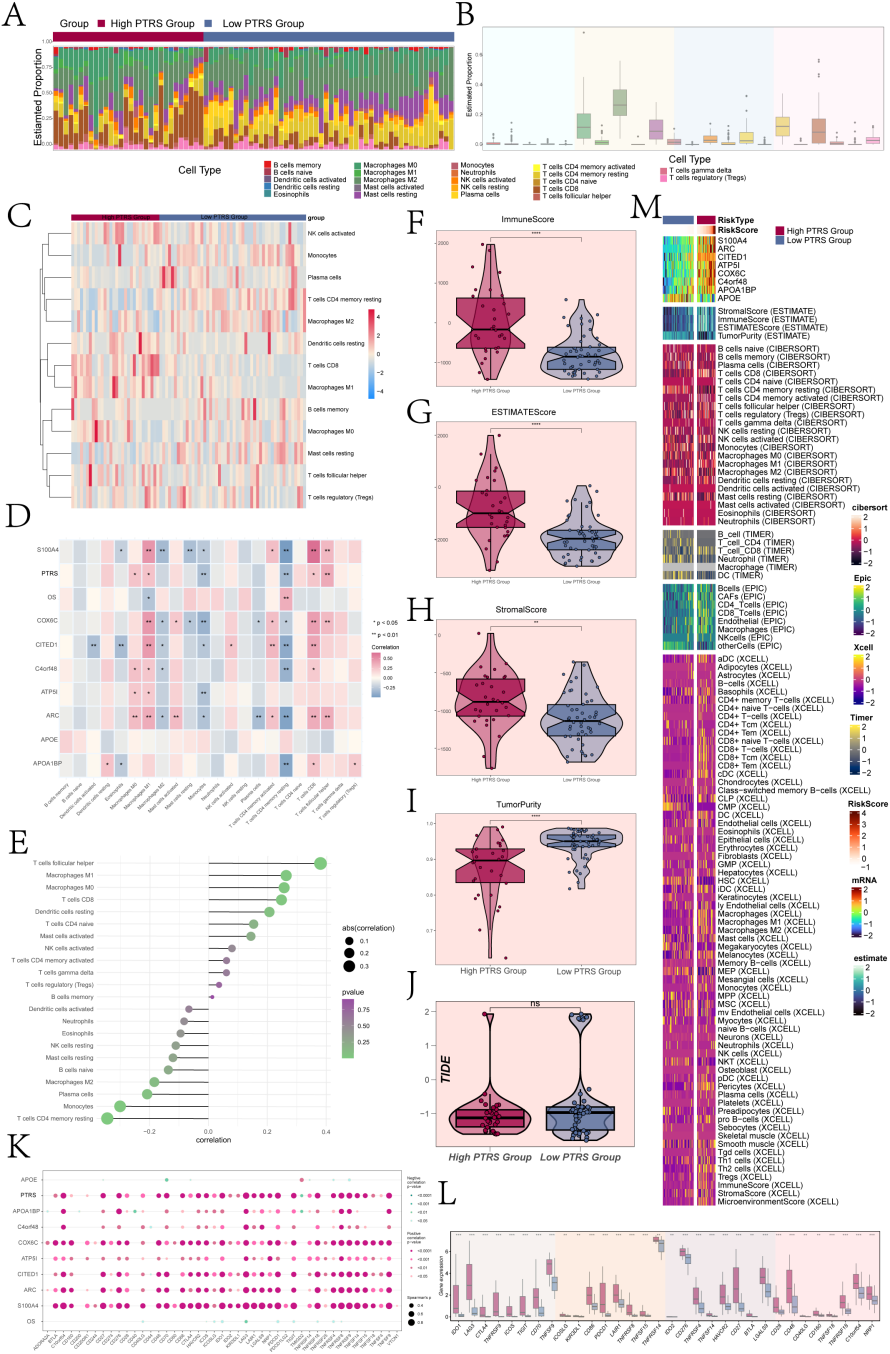
Immune infiltration analysis of different PTRS groups. **(A)** A stacked bar graph illustrated the distribution of 22 immune cells across different PTRS groups. **(B)** A box line graph displayed the proportion of 22 immune cells relative to total cell types. **(C)** A heatmap showed the expression of immune cells with differential distribution in the various PTRS groups. **(D)** A heatmap presented the correlation analysis between prognostic genes, OS, and immune cells in the PTRS model. **(E)** A lollipop plot depicted the correlation between immune cells and PTRS. **(F–J)** Box line plots demonstrated the immune score, ESTIMATE score, tumor purity, and TIDE values for different PTRS groups. **(K)** A bubble plot illustrated the correlation between PTRS-related genes, OS, and immune checkpoint-related genes. **(L)** A box line graph displayed the expression levels of immune checkpoint-related genes. **(M)** Various algorithms, including ESTIMATE, CIBERSORT, EPIC, and Xcell, were applied to calculate immune infiltration in different PTRS groups. *P< 0.05; **P< 0.01;***P < 0.001; and ****P< 0.0001. NS indicated an insignificant difference.

The tumor immune microenvironment significantly influenced tumor progression and affected the response to therapy. To further investigate the heterogeneity among different PTRS cohorts, we analyzed immune infiltration in both Low and High PTRS Groups. **Figures 7A, B** illustrated the distribution of 22 types of immune-infiltrating cells across these cohorts, with macrophages representing a substantial proportion relative to other immune cells. **Figure 7C** detailed the specific distribution levels of various immune cells within the Low and High PTRS Groups.

Moreover, we examined the correlation between immune cell types, prognostic genes, OS, and PTRS within the PTRS model, as shown in **Figure 7D**. M1 macrophages exhibited a positive correlation with most prognostic genes in the PTRS model while being negatively associated with OS. In contrast, resting CD4 memory T cells were negatively correlated with most prognostic genes but positively correlated with OS. **Figure 7E** displayed the correlations between immune cells and PTRS, highlighting that resting CD4 memory T cells were significantly negatively correlated with PTRS, whereas follicular helper T cells showed a significant positive correlation. This suggested their involvement in pro-tumorigenic immune regulatory processes that may facilitate tumor growth or immune evasion. These findings underscored the notion that various immune cell types not only differ in abundance but also maintain a complex dynamic balance in their functional roles within the tumor microenvironment.

Additionally, we calculated the immune stromal scores for the different PTRS groups, revealing that the Immune Score, ESTIMATE Score, and Stromal Score were significantly higher in the High PTRS Group compared to the Low PTRS Group (**Figures 7F–H**). These results indicated a greater degree of immune infiltration and stromal component activity within the High PTRS tumor microenvironment, suggesting a more intricate structural composition associated with tumor progression and treatment resistance. Conversely, the Low PTRS Group exhibited higher Tumor Purity values (**Figure 7I**), implying a simpler microenvironment with less immune and stromal disruption, correlating with improved treatment responsiveness and better prognosis. Notably, TIDE scores did not significantly differ between the two groups (**Figure 7J**).

Finally, we assessed the correlations between immune checkpoint genes and the eight PTRS-related genes, OS, and PTRS, with results depicted in bubble plots (**Figure 7K**). The analysis revealed significant positive correlations between the constructed PTRS and several genes (*COX6C, CITED1, ARC, S100A4*) with most immune checkpoint genes. **Figure 7L** illustrated the expression differences of immune checkpoint genes across PTRS cohorts, showing that most were more highly expressed in the High PTRS Group. We also integrated various algorithms, including ESTIMATE, CIBERSORT, EPIC, and Xcell, to quantify immune infiltration across the PTRS groups, with the results presented in **Figure 7M**.

### Functional enrichment, mutational landscape analysis of different PTRS cohorts

**Figure 8:**
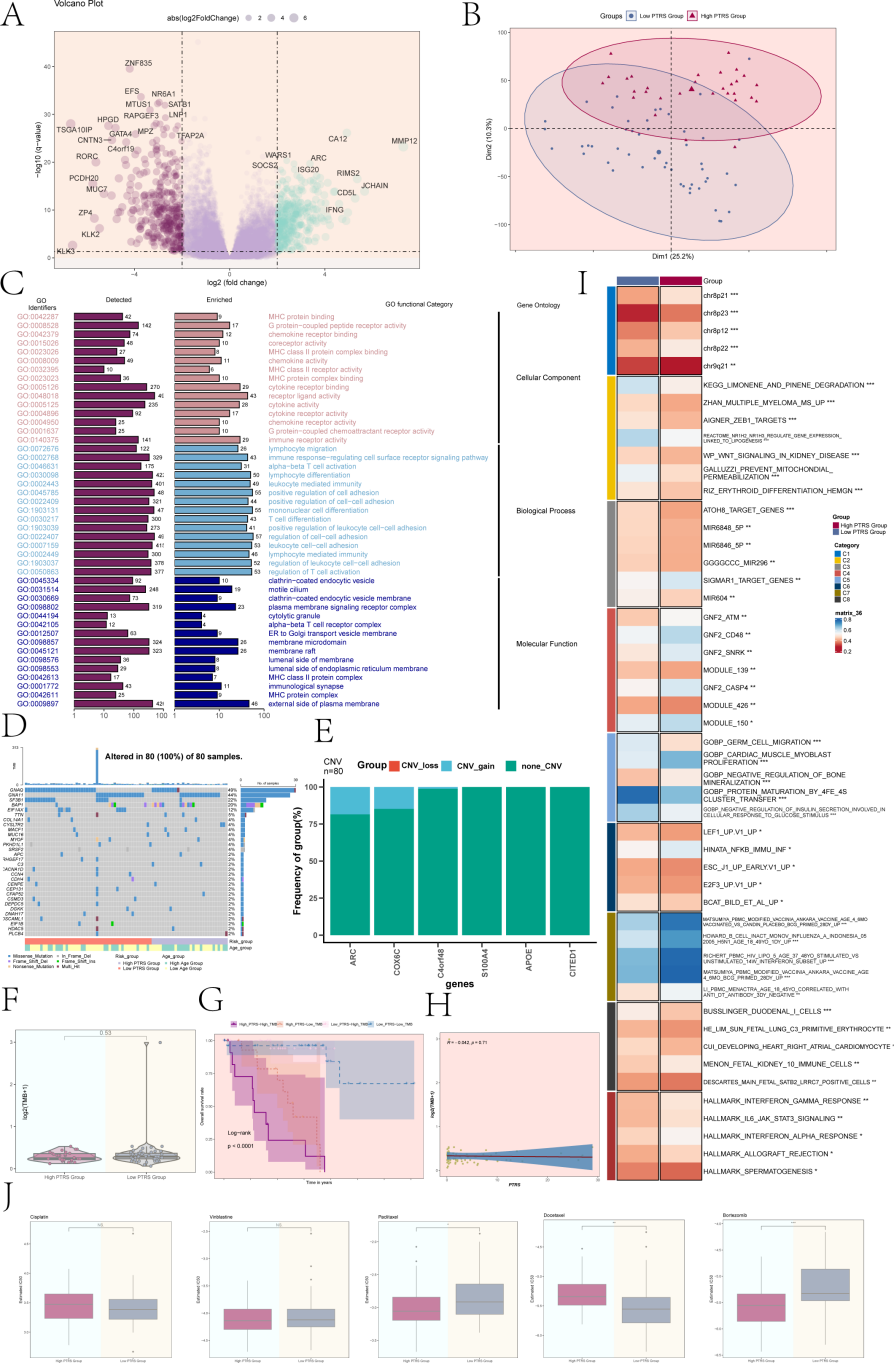
Enrichment analysis, mutation analysis, and drug sensitivity analysis of different PTRS groups. **(A)** A volcano plot displayed DEGs and their distribution across different PTRS groups. **(B)** PCA depicted the clustering distribution of the various PTRS groups. **(C)** Results of GO enrichment analysis was provided for the obtained DEGs. **(D)** A mutation waterfall plot illustrated differences in the top 30 most frequently mutated genes, with the upper column indicating mutation load per sample and the right column showing the total percentage of mutations in these genes. **(E)** A bar graph represented CNV events for selected PTRS model prognostic genes, where green indicated no CNV, red indicated CNV loss, and blue indicated CNV gain. **(F)** Tumor mutation burden (TMB) analysis of different PTRS groups showed no significant difference between the two groups. **(G)** Kaplan-Meier survival analysis results were presented for the High PTRS-High TMB, High PTRS-Low TMB, Low PTRS-High TMB, and Low PTRS-Low TMB groups. **(H)** A scatter plot demonstrated the correlation between TMB and PTRS (R = -0.042, P = 0.71). **(I)** Results of GSVA analysis for different PTRS groups were provided. **(J)** Results of drug sensitivity analysis for different PTRS groups were shown for Cisplatin, Vinblastine, Paclitaxel, Docetaxel, and Bortezomib. *P< 0.05; **P< 0.01; ***P< 0.001; NS indicated an insignificant difference.

We identified DEGs across various PTRS cohorts, presenting the results using volcano plots (**Figure 8A**). These plots effectively illustrated the distribution of significantly up- and down-regulated genes, facilitating the visualization of notable expression differences among the PTRS groups. To further investigate the global variability in gene expression profiles, we conducted principal component analysis (PCA), which revealed that Dim1 and Dim2 accounted for 25.2% and 10.3% of the variance, respectively. This indicated that these principal components capture a substantial portion of the intergroup gene expression differences (**Figure 8B**), reaffirming the significant heterogeneity among the PTRS cohorts.

We subsequently performed Gene Ontology (GO) enrichment analysis on the DEGs, categorizing them into three main classifications: Cellular Component, Biological Process, and Molecular Function (**Figure 8C**). The GO enrichment results highlighted significant associations with several immune-related biological processes, particularly in pathways such as immune response regulation, cell adhesion, and cell signaling at the plasma membrane. These processes were crucial for modulating the tumor microenvironment and immune responses, suggesting that immune regulation may play a vital role in the varying PTRS groups.

The mutation waterfall plot revealed that *GNAQ* exhibited the highest mutation frequency at 49%, followed closely by *GNA11* at 44% (**Figure 8D**). An analysis of CNV events among the PTRS model prognostic genes showed that most genes lacked CNV alterations, although some gains were noted in *ARC* and *COX6C* (**Figure 8E**). However, no significant differences in tumor mutation burden (TMB) values were observed between the different PTRS groups (**Figure 8F**). Based on TMB and PTRS values, the UM cohort was classified into four groups: High PTRS-High TMB, High PTRS-Low TMB, Low PTRS-High TMB, and Low PTRS-Low TMB. Notably, the High PTRS-High TMB group exhibited the worst survival prognosis (P< 0.0001) (**Figure 8G**). The correlation between TMB and PTRS was weak, with R = -0.042 and P = 0.71 (**Figure 8H**).

We further conducted gene set variation analysis (GSVA) utilizing gene sets from the Molecular Marker Database (MsigDB) to elucidate the biological characteristics of the different PTRS groups. The GSVA results, depicted in **Figure 8I**, demonstrated significant differences in multiple biological functions and pathways among the groups. Additionally, an in-depth analysis of GOBP revealed variations in specific biological processes linked to different PTRS groupings (**Supplementary Figure 4A**). **Supplementary Figure 4B** illustrated the Spearman correlation analysis between PTRS and the HALLMARK gene set, further elucidating molecular distinctions among the groups. These findings may provide new insights into the biology of UM and its therapeutic strategies.

To enhance clinical applicability, we systematically evaluated the sensitivity of various drugs in Low and High PTRS groups. We analyzed the differences in semi-inhibitory concentrations (IC50) for these drugs to inform individualized therapeutic approaches. Our assessment indicated that Paclitaxel and Bortezomib displayed higher IC50 values in the Low PTRS Group, suggesting reduced sensitivity to these agents. Conversely, Docetaxel exhibited higher IC50 values in the High PTRS Group, indicating a comparatively weaker effect in this cohort (**Figure 8J**).

### *In vitro* experimental validation

In summary, we selected *CITED1*, the risk gene with the smallest absolute value in the PTRS prognostic model, for in vitro experiments to validate the applicability of the PTRS model in UM. We employed two UM cell lines, MP65 and 92-1, establishing both a negative control group and a *CITED1* knockdown group for comparative analysis.

**Figure 9:**
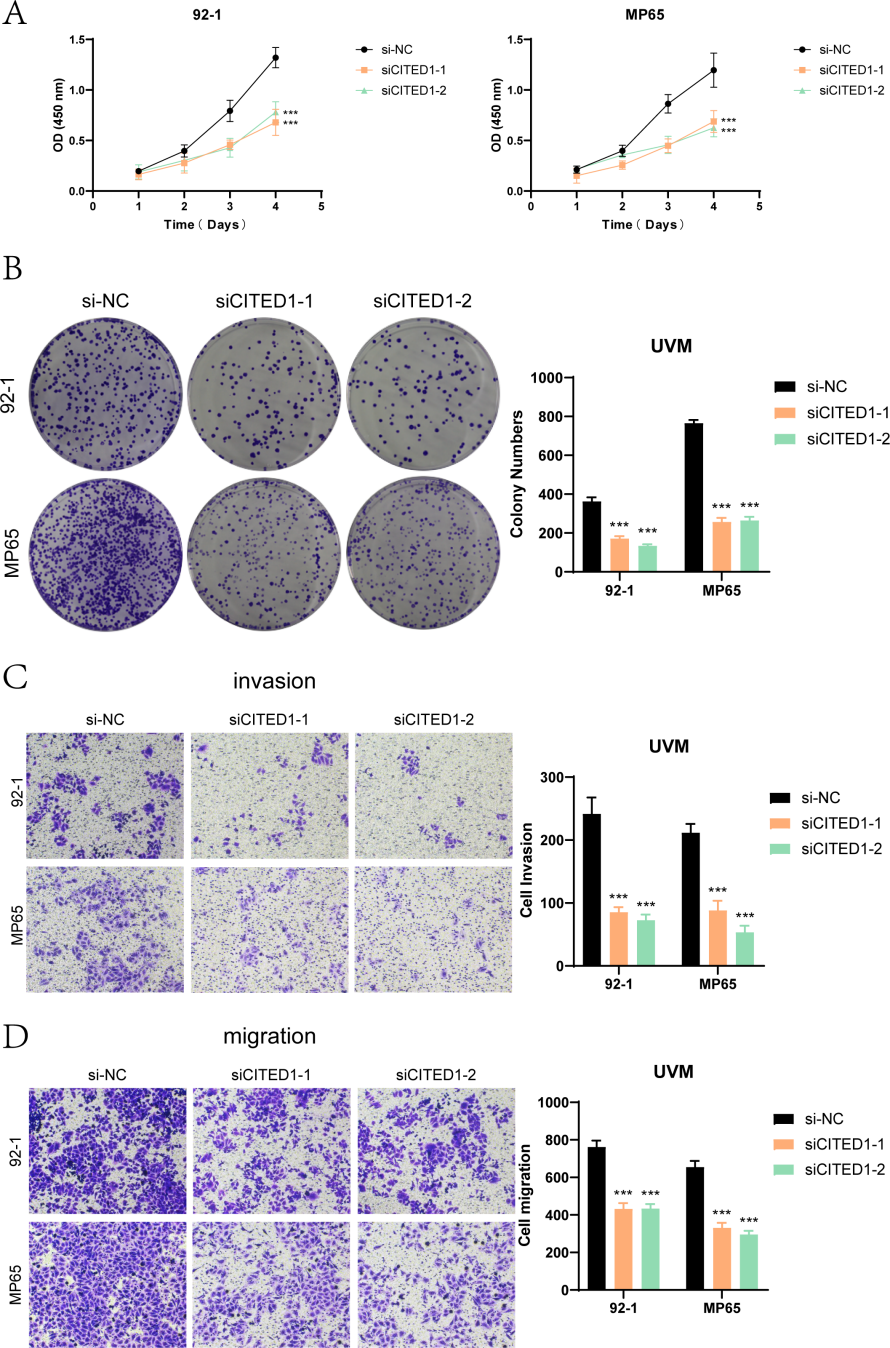
Functional validation experiments of CITED1. **(A)** The CCK-8 assay indicated that CITED1 knockdown resulted in a significant decrease in cell viability. **(B)** The colony formation assay revealed that the number of colonies formed by the CITED1 knockdown cell line was significantly reduced compared to the negative control. **(C, D)** Transwell migration and invasion assays demonstrated that the migration and invasion abilities of MP65 and 92-1 cell lines were significantly reduced following CITED1 knockdown. ***P< 0.001.

Cell viability assays (**Figure 9A**) demonstrated that knockdown of *CITED1* significantly reduced cell viability, as indicated by CCK-8 results. Colony formation assays (**Figure 9B**) revealed a marked decrease in the number of colonies formed by both MP65 and 92-1 cell lines following *CITED1* knockdown compared to the negative control group. In Transwell assays (**Figure 9C, D**), the migration and invasion capabilities of the *CITED1*-knockdown cell lines were significantly impaired.

**Figure 10:**
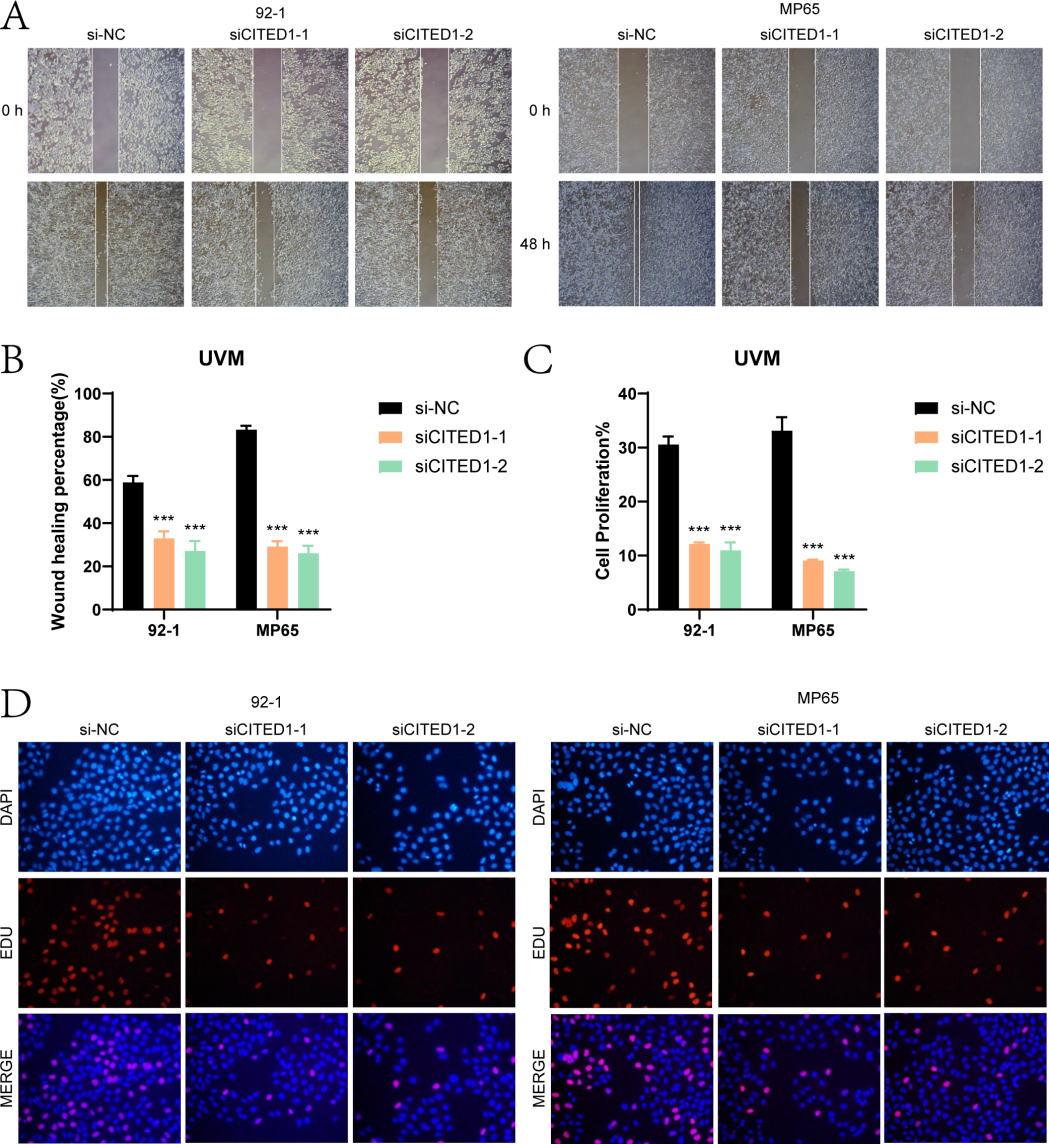
Assessment of wound healing and cell proliferation. **(A, B)** The scratch healing assay indicated that the migration ability of MP65 and 92-1 cells was significantly reduced following CITED1 knockdown. **(C, D)** EdU staining results demonstrated that the proliferation of MP65 and 92-1 cells was inhibited after CITED1 knockdown. ***P< 0.001.

Wound healing assays (**Figures 10A, B**) showed that the scratches in the *CITED1* knockdown groups were considerably wider after 48 hours compared to the negative control, indicating a notable reduction in migratory capacity. EdU staining (**Figures 10C, D**) further corroborated these findings, revealing a decrease in cell proliferation in the *CITED1* knockdown UM cell lines, emphasizing the critical role of *CITED1* in cell growth.

Collectively, our experimental results indicated that knockdown of *CITED1* significantly impaired cell viability, proliferation, migration, and invasion in both UM cell lines. These findings underscored the potential of *CITED1* as a therapeutic target, highlighting its significance in the pathogenesis of UM and supporting the clinical feasibility of the PTRS prognostic model. Thus, targeting *CITED1* and implementing the PTRS model may offer novel strategies and insights for the treatment of UM.

## Discussion

UM is a highly malignant intraocular tumor that significantly affects patient health due to its invasiveness and metastatic potential (61). Although current local treatments—such as enucleation, local resection, and radiotherapy—have shown efficacy (6, 11, 62), controlling tumor metastasis remains a challenge, with many patients succumbing within a year of symptom onset (13). Therefore, understanding the mechanisms behind UM metastasis is essential for developing targeted therapies and predictive models.

In our study, we analyzed the GSE139829 dataset, focusing on single-cell sequencing from eight primary UM patients and three with metastatic disease. This analysis revealed significant heterogeneity among UM cells. We identified eight distinct cell types and confirmed the existence of six subgroups based on marker gene expression. The C5 *PCOLCE*+ TCs subcluster, particularly prominent in advanced tumor stages, may play a critical role in UM progression, supported by its higher Cell Stemness AUC score and CytoTRACE analysis, suggesting it serves as a reservoir for malignant cells.

Cell communication within the tumor microenvironment was vital for UM progression. CellChat analysis highlighted significant signaling between the C5 *PCOLCE*+ TCs and fibroblasts, indicating that this interaction may promote tumor growth through extracellular matrix remodeling. This finding aligned with existing studies demonstrating the importance of tumor-stroma interactions (63, 64). Thus, further investigation of the biological functions of C5 *PCOLCE*+ TCs was warranted as potential therapeutic targets.

Differential metabolic pathway analysis revealed that C5 *PCOLCE*+ TCs exhibit heightened activity in oxidative phosphorylation and glutathione metabolism, which were linked to tumor cell proliferation and survival. This suggested that metabolic reprogramming may confer a survival advantage to this subcluster.

Transcription factor analysis via SCENIC identified HES6 and E2F3 as key regulators in C5 *PCOLCE*+ TCs, with *HES6* showing broad expression across subclusters, underscoring its role in UM biology. The activity of these transcription factors may drive UM cell differentiation and proliferation, providing a basis for future therapeutic studies.

The development of the PTRS model presented a new approach to UM prognosis. Cox regression identified genes within C5 *PCOLCE*+ TCs significantly associated with prognosis, leading to a robust prognostic model validated by high sensitivity and specificity in ROC analyses. Additionally, multivariate Cox analysis confirmed PTRS as an independent prognostic indicator, enhancing clinical decision-making.

The tumor microenvironment profoundly influences cancer initiation, progression, and metastasis (65). Our immune infiltration analysis revealed distinct immune landscapes among PTRS groups. The high PTRS group exhibited complex immune and stromal components, correlating with poorer prognosis. Specifically, M1 macrophages were positively correlated with prognostic genes, while resting CD4 memory T cells showed a negative correlation, suggesting that immune infiltration plays a key role in UM progression and treatment resistance.

Among the prognostic-related genes in the PTRS model, *CITED1* has been identified as a significant high-risk factor. As a key member of the *CITED* family of transcriptional co-regulators, *CITED1* operated as a non-DNA-binding nuclear co-regulator (66, 67). Previous research has demonstrated that *CITED1* overexpression markedly increases proliferation in nephroblastoma (68) and enhances invasion and metastasis in thyroid-like cancer cells (69) Notably, *CITED1* is the risk gene with the smallest absolute value in the PTRS model, leading us to hypothesize that it contributes to the progression of UM. Supporting this hypothesis, our *in vitro* experiments revealed that *CITED1* positively impacted UM cell proliferation, invasion, and metastasis. Consequently, we proposed that *CITED1* may represent a promising prognostic and therapeutic target for metastatic UM.

Overall, this study elucidated the heterogeneity of UM tumor cells and their complex interactions within the tumor microenvironment through systematic single-cell RNA sequencing analysis. The unique biological functions and potential malignant progression characteristics of the C5 *PCOLCE*+ TCs subcluster position it as a significant target for future UM research and therapy. Additionally, the PTRS model provided a novel tool for prognostic assessment of UM patients, indicating promising clinical applicability. Furthermore, the genes within the PTRS model may represent potential therapeutic targets for UM. Future investigations should delve deeper into the specific mechanisms of the C5 subcluster in UM progression and evaluate its potential value in precision therapy.

## Conclusion

This study employed scRNA-seq to analyze tumor subpopulations in uveal melanoma (UM), focusing on immune cell dynamics. We identified that C5 PCOLCE+ T cells, found at the end of the pseudotime trajectory, have high differentiation potential. Through cell communication, metabolic, and transcription factor analyses, we recognized the C5 subgroup as a key population. We developed the PTRS model, which revealed distinct immune landscapes associated with prognosis. The modeling gene CITED1 was validated as a high-risk gene, underscoring potential therapeutic targets for UM.

## Data Availability

The original contributions presented in the study are included in the article/**Supplementary Material**. Further inquiries can be directed to the corresponding author/s.

## Supplementary material

Supplementary Figure 1 UM source data analysis. **(A)** The UMAP figure illustrated the distribution of patient sample sources from 8 primary UM patients (T) and 3 metastatic UM patients (M). **(B)** The UMAP plot depicted the distribution of high-quality cells, categorized into 39 seurat_clusters. **(C)** Cells were annotated into 8 clusters based on distinct marker genes: T_NK, ECs, Tumor cells, Fibroblasts, Photoreceptor cells, RPE cells, Plasma, and Myeloid cells. **(D)** The scale diagram represented the proportion of each cell cluster in each patient sample. **(E)** The scale diagrams illustrated the proportion of each cell cluster from different tissue sources (T, M). **(F)** A bubble diagram presented the top 5 marker genes and their relative expression across the 8 cell clusters.

Supplementary Figure 2 InferCNV analysis. InferCNV analysis results of UM tumor cell subclusters were presented, using ECs as a control. Red indicated high CNV levels, while blue indicated low CNV levels.

Supplementary Figure 3 Results of Cox analysis of key subpopulation genes. Results of univariate Cox analysis were presented for candidate genes of the C5 PCOLCE+ TCs.

Supplementary Figure 4 GSVA analysis. **(A)** T-SNE plots illustrated changes in GOBP pathways and HP gene set activity across different PTRS groups. **(B)** Spearman correlation analysis was conducted between PTRS and the HALLMARK gene set.
