# Supplementary figures and images for: Single-cell RNA sequencing reveals key molecular drivers and immune landscape in uveal melanoma: implications for targeted therapy and prognostic modeling
Source: Front Immunol. 2024 Nov 20;15:1493752. doi: 10.3389/fimmu.2024.1493752 (PMC11615085; doi:10.3389/fimmu.2024.1493752)

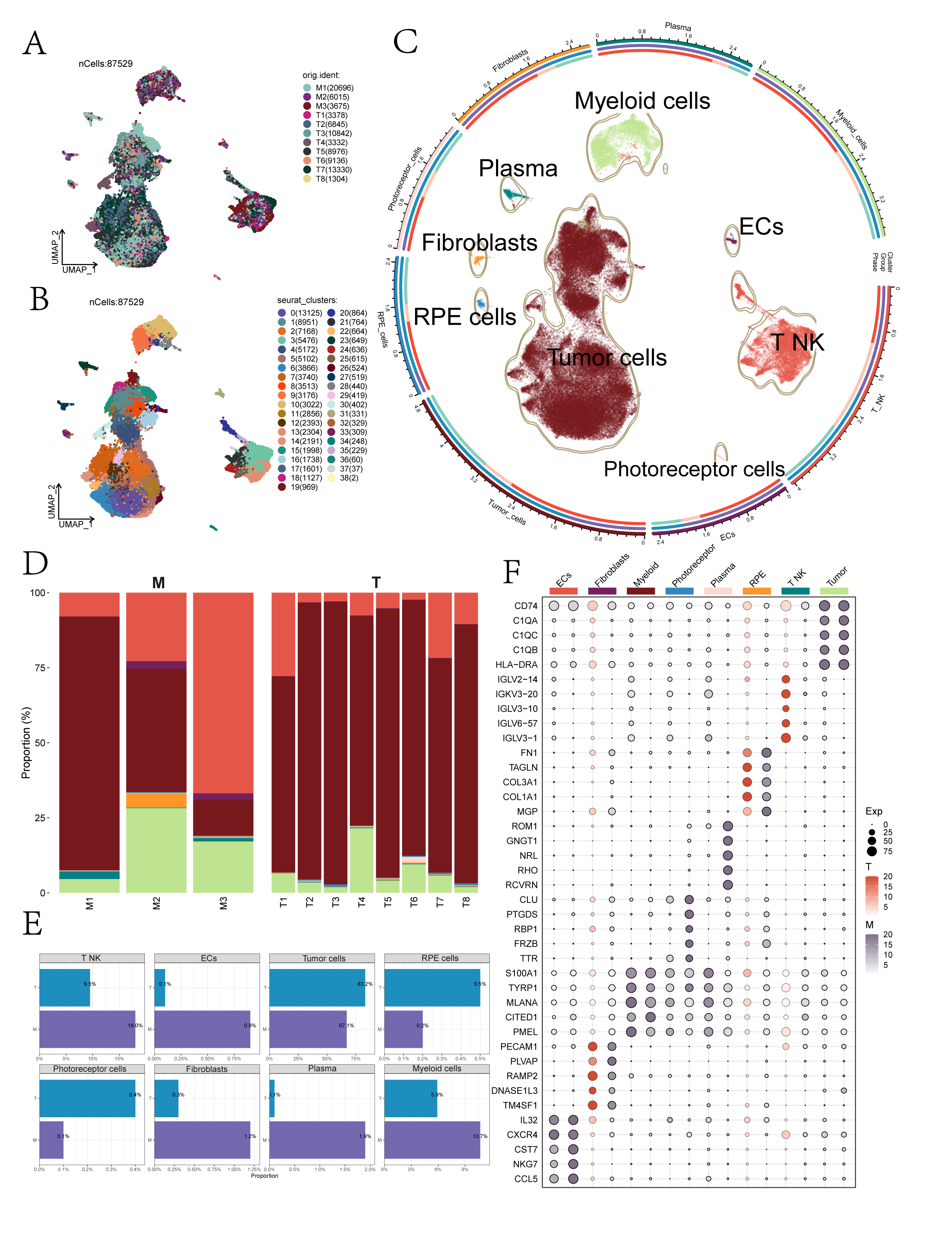

Supplement: Supplementary Figure 1 — UM source data analysis. (A) The UMAP figure illustrated the distribution of patient sample sources from 8 primary UM patients (T) and 3 metastatic UM patients (M). (B) The UMAP plot depicted the distribution of high-quality cells, categorized into 39 seurat_clusters. (C) Cells were annotated into 8 clusters based on distinct marker genes: T_NK, ECs, Tumor cells, Fibroblasts, Photoreceptor cells, RPE cells, Plasma, and Myeloid cells. (D) The scale diagram represented the proportion of each cell cluster in each patient sample. (E) The scale diagrams illustrated the proportion of each cell cluster from different tissue sources (T, M). (F) A bubble diagram presented the top 5 marker genes and their relative expression across the 8 cell clusters. [file Image1.tif]

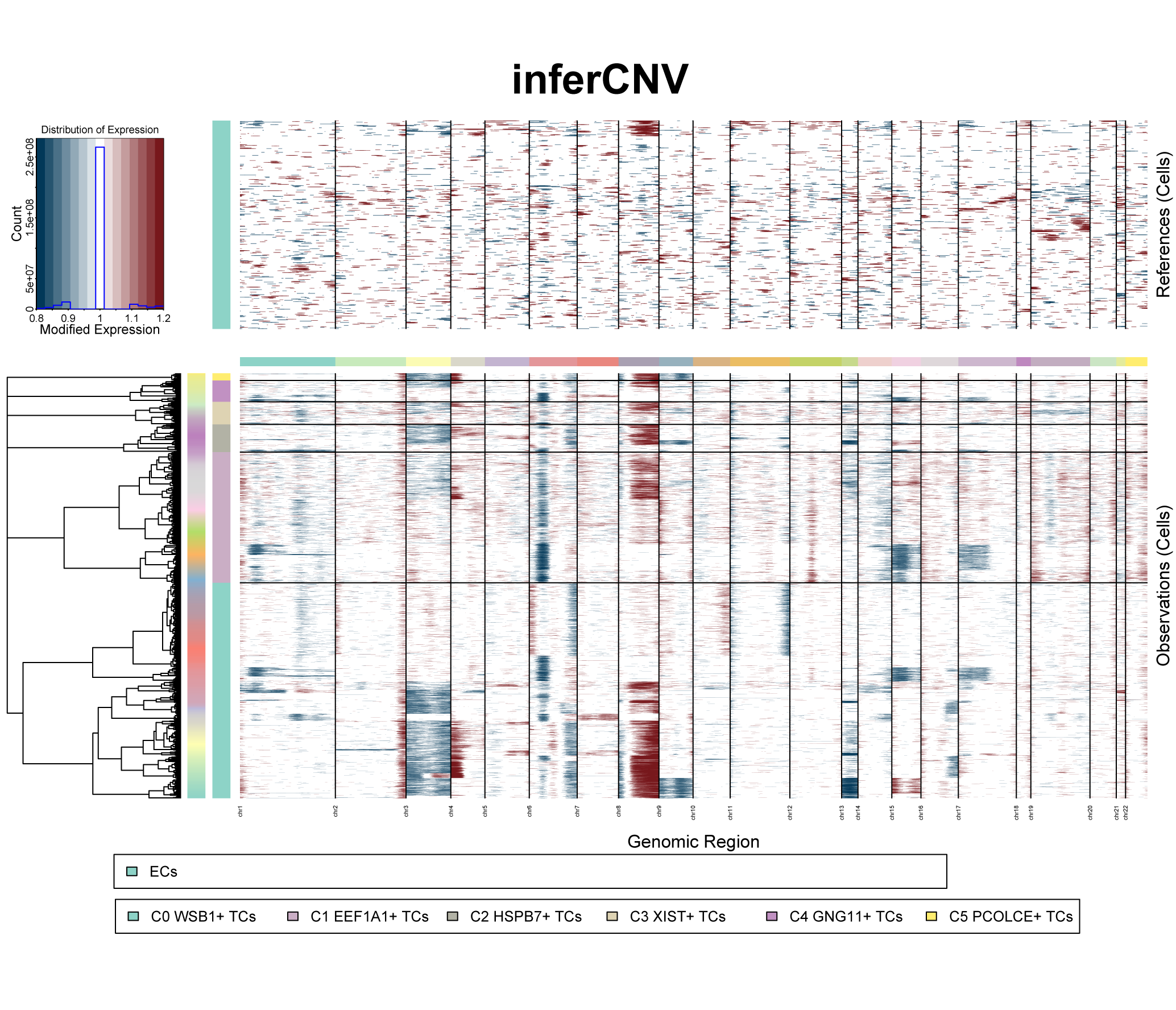

Supplement: Supplementary Figure 2 — InferCNV analysis. InferCNV analysis results of UM tumor cell subclusters were presented, using ECs as a control. Red indicated high CNV levels, while blue indicated low CNV levels. [file Image2.tif]

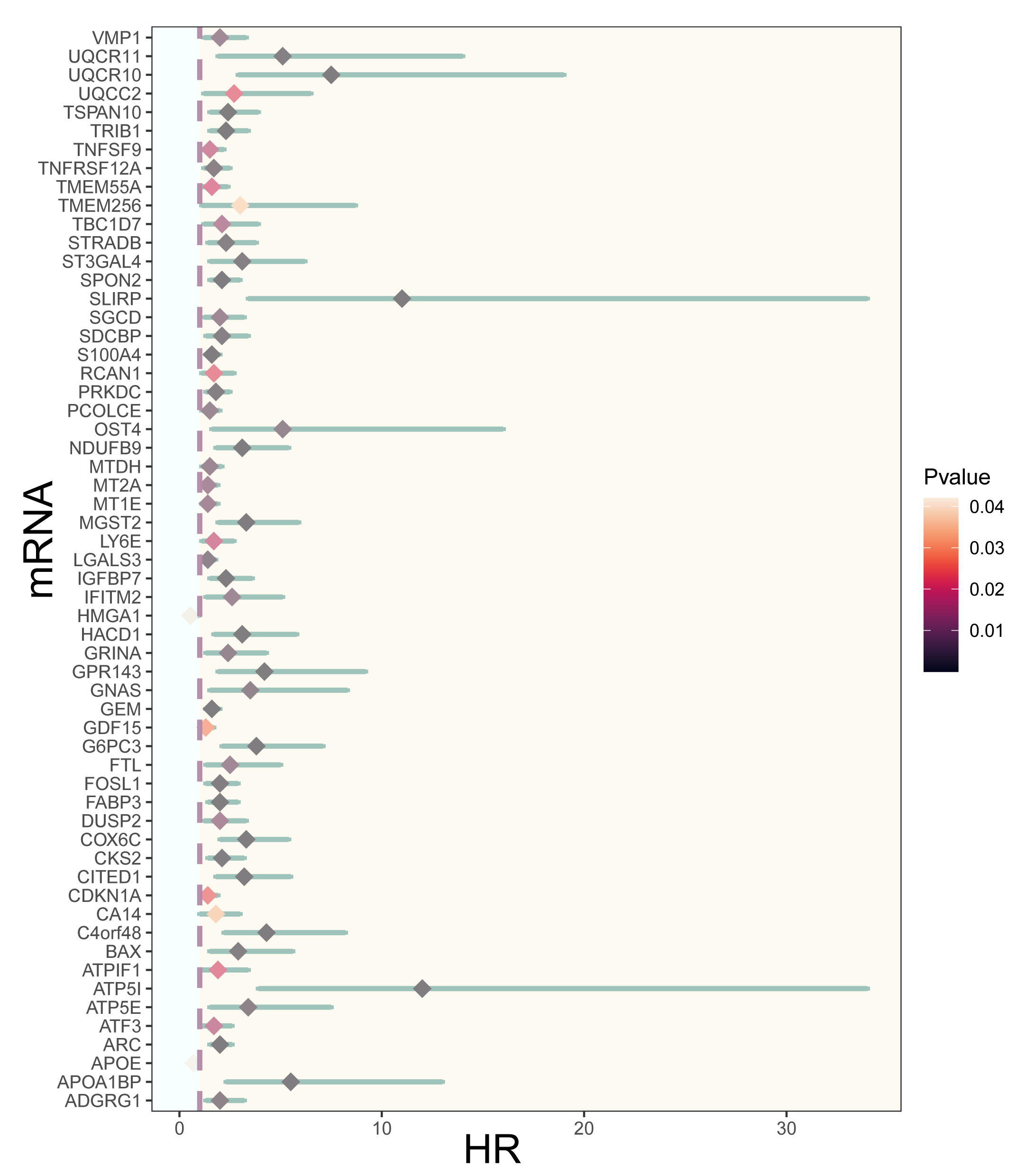

Supplement: Supplementary Figure 3 — Results of Cox analysis of key subpopulation genes. Results of univariate Cox analysis were presented for candidate genes of the C5 PCOLCE+ TCs. [file Image3.tif]

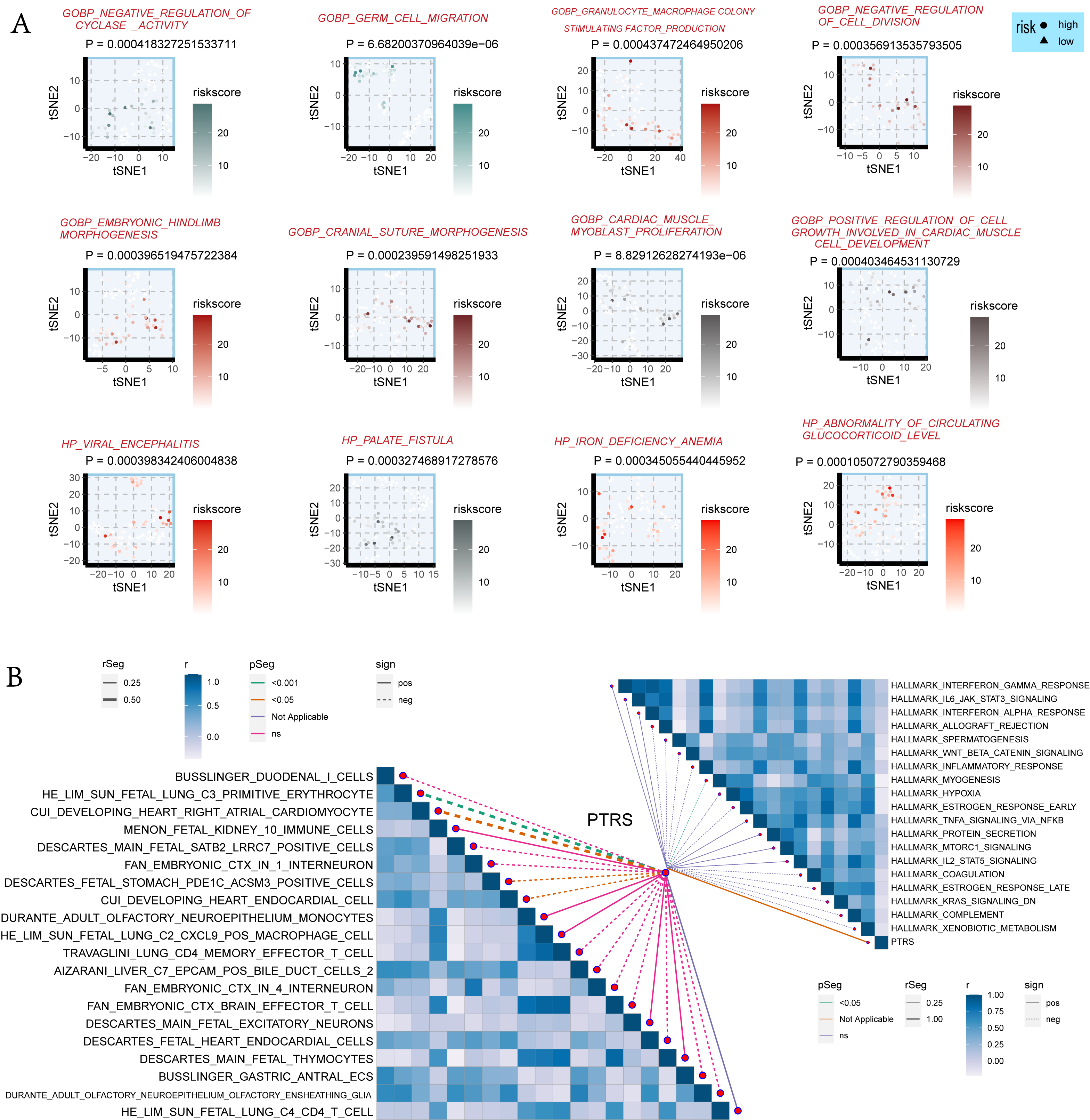

Supplement: Supplementary Figure 4 — GSVA analysis. (A) T-SNE plots illustrated changes in GOBP pathways and HP gene set activity across different PTRS groups. (B) Spearman correlation analysis was conducted between PTRS and the HALLMARK gene set. [file Image4.tif]
